# Effect of bacterial lipase on anaerobic co-digestion of slaughterhouse wastewater and grease in batch condition and continuous fixed-bed reactor

**DOI:** 10.1186/s12944-017-0587-2

**Published:** 2017-10-10

**Authors:** Maha Affes, Fathi Aloui, Fatma Hadrich, Slim Loukil, Sami Sayadi

**Affiliations:** Environmental Bioprocesses Laboratory, LMI Cosys-Med, Centre of Biotechnology of Sfax, BP 1177, 3018 Sfax, Tunisia

**Keywords:** Pre-treatment, Slaughterhouse wastewater, Hydrolyzed grease, Anaerobic co-digestion, Fixed bed reactor, Biogas yield

## Abstract

**Background:**

This study aimed to investigate the effects of bacterial lipase on biogas production of anaerobic co-digestion of slaughterhouse wastewater (SHWW) and hydrolyzed grease (HG). A neutrophilic *Staphylococcus xylosus* strain exhibiting lipolytic activity was used to perform microbial hydrolysis pretreatment of poultry slaughterhouse lipid rich waste.

**Results:**

Optimum proportion of hydrolyzed grease was evaluated by determining biochemical methane potential. A high biogas production was observed in batch containing a mixture of slaughterhouse composed of 75% SHWW and 25% hydrolyzed grease leading to a biogas yield of 0.6 L/g COD introduced.

Fixed bed reactor (FBR) results confirmed that the proportion of 25% of hydrolyzed grease gives the optimum condition for the digester performance. Biogas production was significantly high until an organic loading rate (OLR) of 2 g COD/L. d.

**Conclusion:**

This study indicates that the use of biological pre-treatment and FBR for the co-digestion of SHWW and hydrolyzed grease is feasible and effective.

## Background

The slaughtering industry has become a major component of the agro-food sector in Tunisia. Poultry slaughterhouse wastes are considered as ideal substrates for biogas production, because they usually contain a high concentration of organic contaminants and are rich in proteins and lipids [1]. Therefore, their high biodegradability makes them a good candidate for anaerobic digestion (AD) with the concomitant benefit of energy recovery and waste reduction [2].

Lipids represent an important fraction of particulate organic charge in slaughterhouse wastewater and solid waste that present low biodegradability. Moreover, a high concentrationof lipids in Slaughterhouse wastes may lead to foam formation and sludge flotation in anaerobic digesters as the lipids are adsorbed onto biomass [3]. Also, it may include a reduction in cell-aqueous phase transfer rates and sedimentation hindrance due to the development of filamentous microorganisms [4].

The application of a pretreatment system to hydrolyze and dissolve fats may improve the biological degradation of fatty wastes, accelerate the process and reduce residence time.

A large number of pre-treatment technologies have been suggested to overcome these limitations, such as mechanical, ultrasonic, thermal and alkaline processing. Most of these processes have been reported to improve the efficiency of AD in terms of sludge solubilization followed by improved biogas production [5]. A further promising alternative is the use of the lipase pretreatment that respects stringent environmental regulations and copes with clean and sustainable application of enzymes [6].

Lipases are enzymes that catalyze the hydrolysis of triacylglycerol to glycerol and long-chain fatty acids (LCFA) at the water–lipid interface [7]. These enzymes showed potential applications in hydrolyzing oil and fats in slaughterhouse wastewaters [8].

Several hydrolysis products were tested and Pancreatic Lipase PL-250 proved to be the most efficient in reducing pork fat particle size and increasing LCFA concentration [9].

The use of lipases seems to be the best alternative for treating lipid-rich wastewaters including those from the poultry industry. Dors et al. [10] showed that chemical oxygen demand (COD) removal of wastewater supplemented with different enzymes concentrations was found to be threefold higher than crude wastewater and the production of methane varied from 569 ± 95 to 1101 ± 10 mL per gram of COD for crude and pretreated wastewater, respectively. Due to low hydrolysis rate and inhibition effects, sole degradation of lipid substrate through anaerobic treatment is often not an interest. Thus, a combination of high lipid substrate with other substrates commonly known as co-digestion is a subject of interest. Anaerobic co-digestion has been considered as an effective, low-cost, and commercially flexible approach to improve balance of macro and micronutrients, dilution of inhibitory and toxic substances, increased digestion and stabilization rates, and often an increased OLR [3, 11].

For a high biodegradability, grease waste is considered as a good substrate to be co-digested with low biodegradability substrates [12].

Indeed, Co-digestion of fat, oil and Grease (FOG) with municipal bio-solids at a rate of 10–30% FOG by volume of total digester feed caused a 30–80% increase in digester gas production in two full-scale wastewater bio-solids anaerobic digesters [13].

Similarly, Silvestre et al. [14] co-digested sewage sludge with a trapped grease waste. This study resulted in an increase of biogas production by 138%, and suggested that sludge could become acclimatized to higher FOG loads over time, and that this could be an effective approach for improving fat degradation and reducing the inhibitory effects of LCFA.

Li et al. [15] compared the production of biogas from digestion of Waste Activated Sludge (WAS) with WAS co-digested with FOG using Biochemical Methane Potential (BMP) tests. The biogas production associated to the addition of FOG scored an increase of more than 350%.

The objective of this research was to evaluate the effect of lipase pre-treatment on anaerobic co-digestion of SHWW and grease in batch condition and FBR. A novel *Staphylococcus xylosus* strain was selected for a biological pre-treatment by production of neutrophilic lipase, which could degrade fat and grease present in the lipid waste of poultry industry prior to the anaerobic biological treatment stage.

A Slaughterhouse wastewater was chosen as the liquid substrate because this experiment was part of a larger study evaluating effects of pretreatments on anaerobic co-digestion using a mixture of SHWW and different concentrations of hydrolyzed grease. This study was tested on lab-scale using a FBR with a 3 L capacity and was designed to study the influence of OLR, on the development of fixed-bed reactor technology for wastewater treatment.

## Methods

### Characteristics of the substrates

The wastewater was taken from the Chahia Company, a local poultry processing plant (Sidi Salem, Sfax- Tunisia) and stored at 4 °C until use. The utilized effluents represented a neutral solution (pH = 7), mainly composed of water and organic matter including lipid, phosphorus, nitrogen. The effluent was performed during this study with an average pH of 6.51 ± 0.07, Chemical Oxygen Demand (COD) = 2800 ± 537 mg/l, Biochemical Oxygen Demand (BOD_5_) = 1500 ± 137 mg/l, Total Nitrogen Kjeldahl (TKN) =290 ± 40 mg/l, Total phosphorus (TP) = 59 ± 25 mg/l, Lipid content = 500 ± 230 mg/l, and Total Suspended Solids (TSS) =1565 ± 374 mg/l.

Fatty solid waste samples were collected at the Chahia poultry slaughterhouse waste and stored at 4 °C until use. The anaerobic sludge used as inoculum in biodegradability tests was collected from the sewage plant of Chotrana located in Tunis- Tunisia.

### Microorganism identification, media and culture conditions

The identification of the bacterial strains has been previously determined according to Bouaziz et al. [16]. The strain was kept in LB medium, refrigerated at 4 °C and replicated every 3 months. *Staphylococcus xylosus* was grown overnight at 37 °C and 200 rpm in a liquid medium autoclaved at 121 °C for 2 h containing per liter: 10 g peptone,5 g yeast extract, 5 g NaCl, 1% olive oil; pH 7.0. A 24 h culture of *Staphylococus xylosus* was used as inoculum for lipase production.

### Lipolytic activity assay

The lipase activity in the liquid culture was assayed by measuring the free fatty acids released from mechanically stirred emulsions of triacylglycerols, using 0.1 N NaOH with a pH –Stat (Mertrohm, switzerland). The kinetic assay was performed, in optimal conditions (pH 7.0–7.5 and 37 °C) using olive oil emulsion obtained by mixing (3*30s in a Warring blender), 10 mL of olive oil (Sfax-huile, Tunisia) in 90 mL of 10% Gum Arabic (GA). One lipase unit corresponds to 1 μmol of fatty acid released per minute [17].

### Fat hydrolysis using the lipase producing bacteria *Staphylococcus xylosus*

Hydrolysis of neutral fat of slaughterhouse was tested overnight by the use of *Staphylococcus xylosus*. Cultures used as inocula were performed in Erlenmeyer and incubated in optimal conditions of bacteria strain at 37 °C, pH 7. They were mixed with stirring rod at a rotation speed of 200 rpm over 5 to 8 days. The hydrolysis degree was verified by calculating Soluble COD (SCOD) and lipid contents.

### Lipid extraction and separation

The extraction of grease was determined through solvent extraction with n-hexane. After removal of the solvent by distillation, the sample was dried and weighed and the fat content determined. To separate neutral lipid classes, 1 to 50 μl of neutral lipid extracts or lipid standards at known concentrations were first spotted as 5-mm bands on to a thin-layer silica plate. The elution of lipids was then performed in one step with a hexane/diethyl ether/glacial acetic acid and methanol (78/17/3/2, *v*/v/v/v) solvent mixture, with olive oil and triolein as standards [18]. Following chromatography, the plates were dried at room temperature for 10 min and then immediately sprayed with an iodine indicator. The plates were then placed in an oven to ensure heating at 100 °C for 10 min.

### Biodegradability assay tests

Anaerobic biodegradability batch assays were performed in closed glass flasks with a total volume of 500 mL. It was operated at 37 °C and shaken continuously. Gas volume was measured by syringe-piston displacement. Acclimated anaerobic sludge was transferred to the flasks containing SHWW mixed with different percentages of pretreated grease (25%, 50%, 75%, and 100%) from slaughterhouse poultry waste. Each bottle was fed with an appropriate amount of a substrate and inocula, keeping a VS ratio (VS substrate to VS inocula) at 1:1 in all setups. The tests were carried out in duplicate under batch mode for a period of 50 days. After adjusting pH to 7.2, all the bottles were flushed with a gas mixture of 75% N_2_ and 25% CO_2_ for 3–4 min to supply anaerobic conditions; the temperature was maintained at 37 °C. Control assays were conducted on the inocula, to estimate the volume of biogas and the biodegradability resulting from the fermentation of the organic solids contained in the anaerobic sludge.

The mean values of net biogas production and biogas yields were calculated at the end of each test. The cumulative biogas yield was expressed in terms of mL biogas per gram of COD introduced for different conditions.

### Reactor experiments

**Fig. 1:**
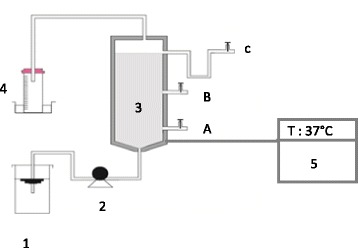
Schematic diagram of the fixed-bed reactor system used for the co-digestion experiments

The co-digestion experiments were performed in a continuously anaerobic fixed bed reactor with a total capacity of 3.5 L and an active volume of 3 L (Fig. 1). A Polyurethane foam cube with a specific surface of 200 m^2^/m^3^was installed in the middle of the reactor as random supporting material. Effluent was continuously loaded into the FBR by using a cycle pump. Biogas volume was monitored daily via the displacement method with water and the corresponding biogas volume was calculated. The biogas was allowed to displace the liquid and the volume of biogas produced was measured either directly by graduation or by the measurement of the weight of liquid displaced [19].

The feed was provided into reactor daily at a fixed time. It was prepared by combining SHWW and HG at a volumetric ratio of 75% and 25% respectively at different OLRs.

COD, TSS, fat content, and pH in the effluent, as well as biogas production were analyzed daily before addition of fresh substrate. At the start-up, the reactor was filled with inoculum for a month to provide a suitable acclimatization of anaerobic biomass prior to the experiments. The general guideline adopted was to increase the OLR when COD removal efficiency and biogas production (± 5%) were constant at high yields for more than three consecutive days.

Before each new feed addition, the same amount of digested effluent was withdrawn from the top of reactor. This operation was conducted in order to prevent washout of biomass from the anaerobic digester especially during treatment at high OLR.

### Analytical methods

The characterization of the influent and effluent stream of the reactor included the determination of the following parameters: pH, Total chemical oxygen demand (Total COD), Biological oxygen demand (BOD_5_) and Total suspended solids (TSS). The pH was measured using a pH meter (OHAUS ST-2100). Total and soluble COD were measured spectrophotometrically after a total digestion with H_2_SO_4_ and potassium dichromate at 148 °C for 2 h [20]. BOD_5_ was determined by the manometric method with a respirometer [BSB-Controller Model 620 T (WTW)]. TSS was determined by weighing samples before and after drying overnight at 105 °C. Fat contents were determined according to the standard methods [20].

## Results and discussion

### Microbial pre-hydrolysis of fats and greases

Bioremediation of lipid-rich wastes by selected lipase producing bacteria was carried out in wastes emanating from slaughterhouse activities. Microbial lipases are metabolically versatile and hence have advantages in diverse industrial processes [21]. The use of enzymes to enhance hydrolysis of macromolecules and enhance the AD process has been investigated for many years especially for fats pre-treatment [2]. In the present study, the biodegradation of 5% of neutral fats from poultry wastes was investigated by using the liquid culture of the lipase producing *Staphylococcus xylosus*.

**Fig. 2:**
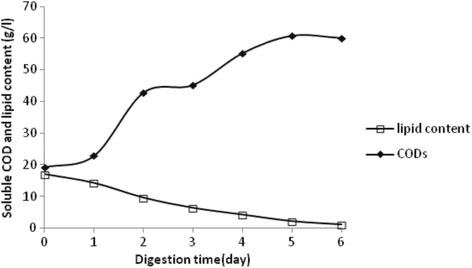
Evolution of the SCOD and the lipid content during time using pre-hydrolyzed grease wastes

Figure (2) illustrates the increasing SCOD and lipid contents obtained during the hydrolysis of poultry waste at different incubations times. In fact, the incubation of grease mixed with LB medium and inoculated with 10% of bacterial culture (with an activity of 15 units/ml) showed that the optimal period for neutral fat hydrolysis is 6 days (Fig. 2).The hydrolysis is influenced by incubation time, and is evaluated by their ability to increase SCOD. In fact, SCOD value increased while the lipid content was reduced from 17 g/l to 1.12 g/l after 6 days of culture.

Similar results were reported for slaughterhouse wastewater samples containing between 2.5 and 3 g/l of lipids pretreated with pancreatic lipase PL-250 for 4 h at room temperature [8]. According to these authors, the proposed pretreatment reduced the average particle size to 60% and increased the free long-chain fatty acid concentrations, indicating partial solubilization of pork fat particles in the slaughterhouse wastewater.

Prasad et al. [21] showed good lipid degradation in slaughterhouse wastewater using *S. aureus* from 25 g/ L to 0.320 g/L and a reduction of BOD_5_ from 3.2 g/L to 0.011 g/l after 12 days of bioremediation.

### TLC analysis after grease hydrolysis by the liquid culture of *Staphylococcus xylosus*

**Fig. 3:**
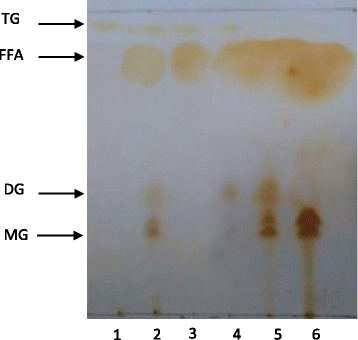
TLC analysis of grease hydrolysis during time by pure culture of *staphylococcus xylosus*. Lane 1: Position of triolein; Lane 2: Olive oil; Lane 3: grease after 1 day of incubation; Lane 4: grease after 2 days of incubation; Lane 5: grease after 4 days of incubation; Lane 6: grease after 6 days of incubation

To confirm the hydrolysis of grease by the liquid culture of *Staphylococcus xylosus*, samples (1 ml) of the reaction medium were collected at different times of hydrolysis, and lipids were extracted and separated by TLC. Figure 3 shows the lipolysis products of grease when this substrate is hydrolyzed by the produced lipase enzyme in the culture of *Staphylococcus xylosus*. The grease is constituted essentially by a mixture of TG (triglyceride), FFA (free fatty acid), DG (diglyceride) and MG (monoglyceride). After 4 days of hydrolysis by *staphylococcus xylosus* culture, we showed the presence of free fatty acid with diglyceride and MG at low intensity. Moreover, spot signals on TLC plate showed clearly that the grease was totally hydrolyzed, and converted into MG and FFA after 6 days of incubation.

### Effect of the concentration of hydrolyzed grease on the biogas production rate

**Fig. 4:**
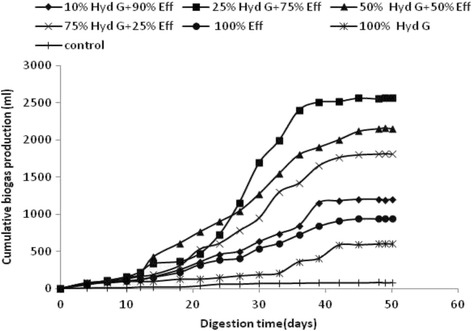
Cumulative biogas production in the different assays of co-digestion of SHWW with HG at different proportions

The cumulative biogas production and kinetic profiles obtained in batch reactors fed with a mixture of untreated slaughterhouse wastewater and different percentages of hydrolyzed grease (10, 25, 50, 75, and 100%) in the presence of the same inoculum are shown in Fig. 4.The biogas production processes ran for about 50 days until biogas production was exhausted. A batch control was also monitored as a serum containing only the inoculum.

The maximum cumulative biogas production from raw SHWW (100% SHWW) and raw HG (100% HG) were 940 mL and 600 mL, respectively giving biogas yields of 0.35 and 0.2 L/g COD introduced respectively.

The biogas yield obtained with 100% hydrolyzed fat is the lowest case after the control (acclimated anaerobic sludge).The inhibition of the anaerobic digestion process was probably due to the accumulation stage of LCFAs, which are important inhibitors on the anaerobic microorganisms, especially methanogens, since the LCFAs adsorption onto the methanogen cells may cause damages to the cell membrane and inhibit the transport of nutrients phenomena through the cell wall [22, 23]. Multiple studies have suggested maximum concentrations of LCFAs, above which anaerobic digesters are likely to experience excessive methanogen inhibition [6, 13].

**Fig. 5:**
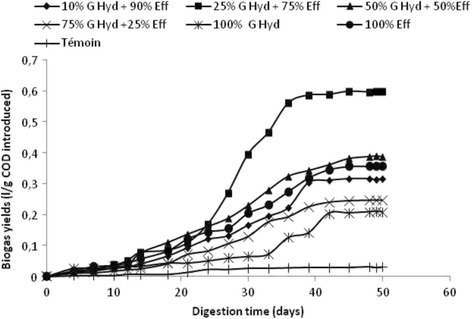
Evolution of biogas yields in the different assays of co-digestion of SHWW with HG at different proportions

The cumulative biogas production at the end of fermentation in serum bottles were 1200, 2560, 2120,and 1810 mL corresponding to the proportions 10%, 25%, 50% and 75% (*v*/v) of hydrolyzed fats, respectively. High biogas production was observed in the batch containing a mixture of slaughterhouse wastewater consisting of 75% SHWW and 25% hydrolyzed grease with a biogas yield of 0.6 L/g COD introduced, this production was twice higher than the crude slaughterhouse wastewater (100% SHWW) (Fig. 5). Similar results were described for the hydrolysis of lipids present in slaughterhouse wastewater using a commercial lipase from *Candida rugosa*. These results show that the pretreated wastewater generated about five times more biogas (500 mL) than the crude wastewater (107 mL) [24].

As for the removal of organic matter in terms of COD, more than 50% were obtained in all cases, with a maximum value reached of 72% for 25% of hydrolyzed fats.

The COD removal was effective owing to the lipid bioconversion into their products (glycerol and LCFA). Mendes et al. [6] showed that the lipid fraction removal by the pancreatic lipase improved organic matter reduction and the biogas formation since pretreated samples were not subjected to sludge flotation, whereas this phenomenon was observed for untreated wastewater.

### Reactor operation

The fixed bed reactor was fed with 25% of hydrolyzed fat and 75% of industrial effluent for 2 months at 37 °C in order to confirm the most efficient mixing ratio and to investigate the performance of the reactor under different OLR.

The start-up period of FBR was relatively about 30 days, noted by an increase of methanogenic Archaea activity as measured by COD degradation and biogas production.

**Fig. 6:**
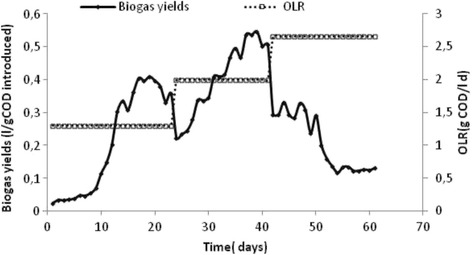
Evolution of organic loading rate and biogas yields during anaerobic co-digestion of SHWW and HG in FBR

The evolutions of OLR and biogas yields, during the anaerobic treatment are presented in Fig. 6.

After the start-up period of the digester, low OLR was maintained (1.3–1.5 g COD/L .d). It should be noted that during this period, acidogenic bacteria use compounds that can be easily metabolized, such as sugars and carbohydrates, whereas methanogens that have a slower metabolism are hindered by the presence of intermediates known as long chain fatty acid (LCFAs). It is reported that LCFAs inhibit metabolism and become the main cause of process instability [25–27].

A maximum biogas yield of 0.4 L/g of introduced COD was registered. An increase of biogas yield was observed after the increase of OLR from 1.5 to 2 g COD/L.d and during the period between 25 and 40 days reaching a yield of 0.6 L/g introduced COD.

By increasing the OLR from 2 to 2.5 g COD/L.d, a decrease in the biogas production was observed. In addition, a low biogas yield (0.3 L biogas/g introduced COD) was registered at HRT of 1.5 day. This decrease can be explained by the effect of the high LCFA concentrations on the anaerobic microorganisms [28].

**Fig. 7:**
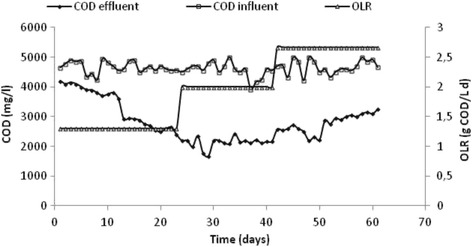
Evolution of the COD inlet and outlet of the reactor and OLR during the anaerobic co-digestion of SHWW and HG in FBR

Results of influent and effluent COD obtained during the period of continuous operation of the fixed bed reactor are shown in Fig. 7.

The variation of influent COD seems to be constant between 4 and 5 g/L. The performance of the fixed bed reactor was affected by the increase of OLR. Therefore, the average of COD removal efficiencies increased from 30 to 53% for OLR of 1.3 and 2 g COD/L.d respectively. For OLR of 2.5 g COD/L.d, the co-digestion process was disturbed. This perturbation in the bioreactor can be explained by the increase of lipid amount in influent [28] which caused an inhibition in growth bacteria.

Consequently, the addition of fats not only allowed higher gas production but also increased the stability of the digestion process. Martinez et al. [29] suggested that the addition of the co-substrate improved the digestion stability and biogas yield via an increase in the OLR from 0.29 to 0.65 gVS L^−1^ d^−1^ for the mesophilic system. In the studies realized by Luostarinen et al.[12], the co-digestion of grease trap sludge from a meat-processing plant and sewage sludge increased specific methane production in reactor experiments (maximum 463 m^3^/tVS added) compared to digestion of sewage sludge alone (278 m^3^/tVS added).

## Conclusion

The co-digestion of hydrolyzed grease and slaughterhouse wastewater was successfully performed in batch and reactor conditions. The best biogas yield and biodegradability were obtained at 25% (*v*/v) of HG. This proportion was tested in semi-continuous operation. Results confirmed that co-digestion of 75% SHWW with 25% HG (v/v) in anaerobic FBR yields the best biogas yields and process stability until an OLR of 2 g COD/L .d, by acquiring a stable environment, nutritional balance in the anaerobic micro-ecosystem and reducing inhibitory effect of LCFAs by the application of microbial pre-treatment during hydrolysis. This is due to the increase in the transportation rate of soluble substrate into biomass to induce biogas conversion.

## Abbreviations

AD: Anaerobic Digestion
COD: Chemical Oxygen Demand
HG: Hydrolyzed grease
HRT: Hydraulic retention time
LCFA: Long-chain fatty acids
OLR: Organic loading rates
SCOD: Soluble Chemical Oxygen Demand
SHWW: Slaughterhouse wastewater
